# Upstanding Youngsters: Ivorian Scouting and the Manufacture of a Subordinate Citizenship (1937–1979)

**DOI:** 10.3389/fspor.2020.568013

**Published:** 2020-10-08

**Authors:** Claire Nicolas

**Affiliations:** Faculté de sciences sociales et politiques, University of Lausanne, Lausanne, Switzerland

**Keywords:** scouting, Côte d'Ivoire, sport, youth, independence, gender

## Abstract

This research focuses on Ivoirian scouting from its colonial implementation in the late 1930s up to the late 1970s, under Félix Houphouet-Boigny's regime. Using a novel perspective, it highlights the gender lines of scouting youth training in West Africa. Furthermore, this paper argues that understanding this history of Ivoirian youth through the lens of the scouting movement allows us to articulate youth governance between the colonial and postcolonial era, notably in order to understand the political and moral subordination of Ivoirian youth during the twentieth century. This research is based on archives collected in France, Côte d'Ivoire and Switzerland as well as biographical interviews conducted in 2016.

“In my community, the Scouts, the vast majority are children of civil servants. Because first of all, they are well-dressed. They march. It gives the impression of order and discipline. So, everyone was proud to see their child in these ranks!”^1^.

When, in 2016, a former Ivorian Boy Scout recalled what attracted him to Scouting 50 years earlier, as a teenager, he emphasized its playful aspects and the camaraderie it involved, but also placed great emphasis on the privileged social status of young Scouts in Ivorian society^2^. At the time, the Scouts largely came from the privileged ranks of the State bourgeoisie that managed the country (Fauré and Médard, 1982), and the civil service embodied a form of social success. At the same time, the values of order and discipline were praised by the *Parti Démocratique de Côte d'Ivoire* (Democratic Party of Côte d'Ivoire, hereafter PDCI), which now ruled the country. In particular, young people had to be disciplined and subordinate themselves to the values promoted at the State level.

The career of this former Scout, like that of his comrades, is part of the political and social history of Ivorian youth and can be understood as such from a social and political perspective. And one perspective within the field of contemporary African history is particularly enlightening. For scholars such as Mbembe (1985), young people are social and political juniors who were excluded or instrumentalized by many States in sub-Saharan Africa before and after independence. This paper will thus argue that Scouting, as a youth movement of European origin promoting loyalty, morality, and physical exercise, allows us to understand the ambivalence of the relationship between young people and the colonial, then post-colonial African States they live in, through the example of Côte d'Ivoire. Here, the Ivorian case helps us illuminate how the perspective of social and political history enables us to understand how young people and their engagement in physical activities may be associated with a public social engineering project.

Scouting, a movement established by Robert Baden-Powell at the beginning of the twentieth century, aims to train young people from a moral and physical perspective. Conceived as an imperial practice of military inspiration for young boys in the United Kingdom (Block and Proctor, 2009), the movement was very quickly taken up by girls (Mills, 2011) before spreading abroad, in Europe (Kergomard and François, 1983; Laneyrie, 1985; Cholvy, 1999; Prigent, 2011) and all over the globe, notably in the French and British empires (Parsons, 2004; Johnston, 2016; Fossard, 2017; Wu, 2019). In the case of the latter, the historian Timothy Parsons has shown how Scouting, as an institution promoting social stability and loyalty to the Empire, is a faithful ally of the colonial administration. However, French-language works have recently highlighted the emergence of fault lines in this loyalty, notably the subversive role played by Scouting in the French Empire at the time of the struggle for independence (Bancel et al., 2003; Krais, 2018).

The rise of Scouting in Côte d'Ivoire took place within a particular context. A colony relegated to the margins of the Afrique Occidentale Française (hereafter AOF)^3^, Côte d'Ivoire became one of the leading economic powers in the region after gaining independence in 1960, while the country's economic development was based on State capitalism and cooperation with France (Bamba, 2016). As such, specialists in the political history of Côte d'Ivoire have highlighted the determination of President Félix Houphouët-Boigny to offer a development model based on cooperation with the former French colonial power, as well as the iron fist with which he ruled the country (Fauré and Médard, 1982). In the early 1960s, among other things, the President eliminated his opponents within his own party, in particular removing the head of the Marxist *Jeunesse du Rassemblement Démocratique Africain de Côte d'Ivoire* (Youth of the African Democratic Rally of Côte d'Ivoire) in 1964^4^. During the following decades, he came under increased criticism, particularly from young Ivorian graduates who pointed out his poor economic management and the control he exerted over their organizations (Chazan, 1976; Proteau, 2002). In response, in 1968, he dissolved the student union heading this protest and repressed student demonstrations^5^. However, examining this history of Ivorian youth through the lens of the Scouting movement allows us to articulate another aspect of this opposition between student sedition and State repression. More specifically, this paper focuses on what Jean-François Havard refers to as “socialization in a subordinate political assignment”^6^ (2009, 316), meaning that the “Fathers of Independence” made sure that “social juniors” were also “political juniors,” setting up specific control procedures in order to better mobilize them for their own benefit on the one hand, or exclude them from the higher spheres of the political arena on the other. Scouts were not completely excluded from the political game, however: rather, they were confined to a marginalized position from which they could be instrumentalized by the Ivorian State in response to various forms of contestation. Thanks to their sense of duty, loyalty, willingness, and respect for hierarchies, Scouts ideally embodied the figure of the “new man” (Bromber and Krais, 2018), the archetype of the new postcolonial citizenship, and were at the vanguard of the modernization process—albeit subordinate to political power.

If historiography offers avenues of analysis to understand this subordinate political assignment, it remains incomplete. First, the focus on Ivorian student rebel youth put forward by political historians of Côte d'Ivoire does not allow analyses to take into account the nuances of a diverse younger generation, which certainly was subversive in parts, but also included disciplined and loyal individuals. Political discourses might define “youth” as a natural and unambiguous stage of life, either feared for its tendency for deviance, misconduct, and rebellion, or praised for its enthusiasm and creativity. However, the reality is far more complex, and scholars of youth have shown the importance of an intersectional approach to qualifying young people (Bantigny and Jablonka, 2009). Secondly, academic works on Scouting in Africa tend to focus on the colonial era (Bancel, 1998), and those that focus on the post-colonial decades tend to limit themselves to issues related to cooperation (Harang, 2010) or development (Koné, 2017). However, Katrin Bromber's publication (Bromber, 2018) on the place of Scouting in the formation of an Ethiopian “new man” and, more generally, scholarly works on socialist youth movements in Africa (Burgess, 2005; Ahlman, 2017; Nicolas, 2017) offers new lines of inquiry in terms of the training of young people by the state. Thirdly, the sub-field of “Scouting Studies,” in line with Sub-Saharan Sport Studies, remains largely andro-centered (Sikes and Bale, 2014), in spite of some recent works on colonial Girl Guiding (Krais, 2019; Wu, 2019). Following this innovative avenue of research, understanding Scouting more widely through the prism of the Girl Guides' moral salvation program helps throw light on the ways in which the Ivorian Scout movements developed as training institutions dedicated to an ideal younger generation.

This analysis uses multi-situated data (Nicolas, 2019a). Indeed, archives related to the Ivorian Scouting movements are scattered. Some may be located within the archives of public authorities, as they gathered intelligence on youth activities. One may also go through private archives (either held in public archive centers or by organizations themselves), which are scattered from Côte d'Ivoire to France and Switzerland. In Côte d'Ivoire, I thus accessed the archives of the Ministry of Youth and Sports and the library of the *Institut National de la Jeunesse et des Sports*. There I was able to go through the archives of the *Association des Guides de Côte d'Ivoire*, in the collection of the Frenchwoman Marcelle Piault (1960–1985). In order to explore the premises of Scouting during the colonial era, I collected archival documents in the *Archives nationales d'Outre-mer* in Aix-en-Provence, France (in particular the *Bulletin de la Côte d'Ivoire*) and from the *Archives nationales du Sénégal*, in the Afrique Occidentale Française (AOF) collection (in particular the political archives). In addition, the *Eclaireurs et éclaireuses de France* association collection in the *Archives départementales du Val-de-Marne* (France) provides access to the private collection of two French Scouts linked to Côte d'Ivoire: René Dumeste (1952–1954) and Charles Boganski (1963–1979). Moreover, within the archives of the *Scouts et Guides de France* association in Paris, it is possible to browse through Scouting magazines published in AOF. Finally, in the Swiss Federal Archives, I went through the collection of the *Fédération des Éclaireuses Suisses*. The archive documents a cooperation project with the Ivorian Girl Scouting movements (1968–1975). However, as the archives are scattered, fragmented and sometimes unavailable, some data are lacking. In particular, while we may plausibly infer^7^ that there were around 200 young Boy Scouts and Girl Guides in the 1950s and several thousands in the 1970s, it is impossible at present to carry out a precise quantitative analysis of the movement over the decades. In 2016, in collaboration with the Ivorian Scouting specialist Gilbert Koné, I conducted biographical interviews with 11 former Ivorian Scouts and Girl Guides who participated in the movement during their youth, between the 1950s and 1970s. Cross-examination of these archives and interviews allow us to contrast the perspectives of the colonial and independent public authorities on the one hand, and Scout leaders and former young members of the Scouting movements on the other.

## The Colonial Origins of Scouting: Toward a Hierarchical Brotherhood (1937–1959)

Scouting movements were established in Côte d'Ivoire during the colonial period. They were first imported from France by French citizens, either members of the colonial administration or missionaries. Catholic, secular and Protestant movements were closely associated with the colonial civilizing mission, particularly with a view to training young Ivorian schoolchildren for their future role as auxiliaries to the colonial State. While French Scouting massively took off in the 1920s, it was not until the end of the 1930s that the first troops were founded in the colony. As Scouting aimed at training a social vanguard, the movement found a fertile ground in the colonial “civilizing mission.” And, far from enrolling large swathes of Ivorian youth, the movement grew from Catholic schools and churches, reaching an educated urban youth in the Southern region.

The first recorded troop in the colony was founded in 1937 by a metropolitan missionary Father and a leader (a French female nurse from Dakar), in the parish of the African district of Treichville, in the south of Abidjan^8^. The Catholic *Scouts de France* movement was closely associated with the evangelizing mission of the Church. Troops comprised of young African boys and led by white fathers appeared between the late 1930s and the early 1940s and were integrated into churches and Catholic schools. These troops were concentrated in urban centers in the southern areas of the country (Agboville, Abidjan, Bingerville). It was only under the Vichy regime that the secular *Éclaireurs de France* and—to a lesser extent—the Protestant *Éclaireurs unionistes* established troops in the colony^9^. Throughout the 1940s, the secular movement was not very present in Côte d'Ivoire. It was only from 1952 onwards that the movement really took off^10^. Before this date, Bancel (2009, p. 149–150) counted 150 Catholic Scouts and 50 secular Scouts in colonial Côte d'Ivoire. In this respect, the territory was different from the other colonies in Afrique Occidentale Française, most of which were committed to the cause of secular Scouting. Finally, despite the involvement of a handful of Catholic Girl Guides in Abidjan from the 1940s, Girl Scouting was far less developed^11^. These organizations had neither the support of the clergy nor that of the education services. It was only in 1957 that a permanent company was created at Bingerville's Girls secondary school, soon followed by units supported by Catholic missionary sisters in Abidjan, then throughout several girls' schools in the Southern region^12^.

All in all, during the last three decades of colonization, the Ivorian Scouting movement was mainly monitored by the missionary Catholic clergy, either through Catholic schools or churches. One may frame this distinction in terms of the special place occupied by Côte d'Ivoire, at the periphery of the AOF. On the one hand, its primary school enrolment rate was among the lowest in the French colonies^13^ and, on the other hand, elite secondary schooling was concentrated more than 2,000 km away in Dakar, Senegal (Jézéquel, 2003; Barthélémy, 2010). As a result, secular Scouting, set up within secular colonial schools, remained scarce in Côte d'Ivoire until the end of the Second World War and the subsequent rise in school enrolment throughout the territory. Furthermore, Catholic Scouting remained limited to the urban areas of the coastal South. Here, colonial control was strong and stable, in contrast to the forest and northern areas, where the military and political administration was both more scarcely distributed and violent (Gendry, 2020). Meanwhile, in colonial urban centers, colonial Scouting groups remained mainly embedded within Catholic institutions and public administrators favored their development. This attitude was consistent with the ideology of a civilizing mission that officially underpinned France's imperial conquests from the end of the nineteenth century. Boy and Girl Scouts were to become a positive model, the faithful civilized youth opposed to a rebellious territory. Indeed, not all Africans should become involved in Scouting, but rather an educated elite. According to administrators who favored French Scouting, it thus provided “a magnificent example of French unity, [but it remained] an elite movement reserved for the training of leaders”^14^. These “leaders” were indeed to become the auxiliaries of the colonial administration (Jézéquel, 2007), as clerks, nurses or teachers. Eventually, they were to become colonial middlemen, led astray from a supposed African savagery, while remaining subordinate.

However, as Tony Chafer has recently pointed out, even if the “civilizing mission” discourse aimed rhetorically at the cultural and political assimilation of an educated elite, it was in fact more of a way to “legitimize a form of segregation between French people and Africans” (Chafer, 2018, p. 4). In this perspective, appeals were constantly made to hierarchy as far as the “small indigenous elite” that populated the Scout movements was concerned. The words of the AOF's Director General of Political, Administrative and Social Affairs in 1942 echoed this ideology, when he defined Scouting as follows:

“A true brotherhood, in which we play the role of elder brothers, is born between white and black Scouts jointly serving the same ideal. It is a hierarchical brotherhood at the top of which our homeland, France, radiates”^15^.

The French elder brothers were to raise their younger brethren toward the French homeland, while keeping them in subordinate positions. As such, Scouting had to enable young boys to forge a virile will, worthy of France, and to emerge from the “atavistic instincts of their race”^16^ so that they could be energetic, willing, adventurous, pure, and fraternal. The colonial movements here echoed the moral enrolment program that was created during this period by the Vichy regime for its metropolitan youth (Prêtet, 2016). The subordinate status of the African Scouts was thus recalled and asserted by the head of the colonial administration.

In practice, for young Ivorian schoolchildren involved in Scouting, learning was based on physical and sports pedagogy, which complemented school. Following the example of Baden-Powell's initial model, large-scale games in nature were intended to promote their moral and civic training. Thus, Scouting brought together the elite “of those who [knew] how to promise and show[ed] themselves capable of keeping their commitments”^17^ (Denis, 2003, p. 199), while Scouting trials and exams provided proof of such moral fiber.

For instance, the activities organized in Bouaké (Central Côte d'Ivoire) by Catholic and secular Scouts in the 1950s provide an insight into how the Ivorian Scouts put in place this moral and physical training project. The *Scouts de France* organized bivouacs and, during these camps, centered their activities around religious ceremonies. Thus, for example, the leader Dorothé Akpovo (a teacher) and his troop *Saint-Michel* (protected by the patronage of its eponymous saint) camped for a few days in a village near Bouaké over Christmas 1955. On this occasion, the young boys made a campfire, sang, danced, played, and exercised. They also took advantage of this opportunity to conform to the social doctrine of the Church: they attended baptisms, performed “multiple services” in the village and prayed every day^18^. In the same place, in the same year, the *Éclaireurs de France* engaged creatively with the Catholics to organize appealing activities. For example, the Bouaké patrols went boating and swimming by regularly going up the Bandama River with canoes and inflatable rafts over several dozen kilometers. For both Catholic and secular Scouts, physical activities and games were associated with discussions on morality and socially beneficial activities.

The learning of morality and civics also touched on the private lives of young boys. Indeed, the French colonial administration sought to order family life by exalting the centrality of a domestic ideal inspired by the European bourgeoisie. The family unit was to allow for the reproduction of heritage on the one hand and the reproduction of social values through the education of children by women on the other (Barré, 2019). The promotion of European conjugality involved, among other things, movements interested in “educated” organizations, such as Scouting. For example, discussions among the Scouts were often devoted to conjugality and the duties of young Scouts, such as conjugal duty, the founding of a home, or saving money^19^. The following excerpt from the AOF Catholic Scouting journal gives a good example of such advice:

To get married, “it is necessary firstly that one's studies be completed or about to be completed, and secondly that the financial conditions in which the young man and the girl live be such that they can safely assume the responsibility of a home. It is also preferable that the spouses practice the same religion.” ^20^

However, even beyond the Boy Scouts movements, as family and conjugality became central issues for the civilizing enterprise of the colonial State, it was mainly the Girl Guides that were targeted in this perspective.

Through her work on Lagos (Nigeria), George (2014) has highlighted a double movement of changes to social policy during the 1940s in West African colonial cities. She has shown how girls were then constituted as a category “to be saved,” with moral salvation becoming a political discourse participating in the reorganization of colonial political power after the Second World War. Moreover, Gendry (2020) recently put forward in her thesis on AOF's judicial space that such injunctions were not the same in urban and rural areas. For colonial legislators, non-respect of Western moral standards was tolerated in the “bush” on the basis of a “savagery” and “amorality” that was supposed to be peculiar to Africans (and in particular a sexuality that was expressed outside the rules of European decency). In contrast, in cities, proximity to Europeans required (again, in the eyes of legislators) a stronger acculturation to such moral standards. Consequently, young African girls living in colonial cities were all the more the object of their attention, following the model of European charities (Belliard, 2004).

And indeed, the Girl Guides of Côte d'Ivoire movement was built as a bulwark against the moral dangers of urban life. Thus, in 1943, French leaders of the French colonial Girl Guides movement deplored the “feverish and unethical atmosphere of big colonial cities” and, to remedy this, proposed the establishment of “women's restaurants and street surveillance along with morality propaganda”^21^. While no actual traces of these surveillance projects have been found, restaurants and non-mixed hostels were established by the Girl Guides movement in the colony^22^. In order to exert a beneficial moral influence on the girls, leaders thus offered to integrate them into the private sphere and encouraged them to leave the public space. Once again, the desire to control colonial sexualities was resurfacing (Stoler, 2002). However, it is not the sexuality of young men that was targeted, nor possible sexual assaults, but rather the sole behavior of Ivorian girls.

However, despite this action in accordance with the principles of the civilizing mission, the Girl Guides movement did not benefit from the blank check granted to the Boy Scouts movements by the colonial authorities. The leaders of the movement were constantly looking for funding. But in 1944, the AOF's General Secretariat of Youth refused to help them:

The Girl Guides movement must only “emancipate [women] very slowly and there would be no question, apart from the exceptions that will confirm the rule, of training parallel to that of boys as we understand it. Since women are the means of work and the instrument that perpetuates life in the eyes of Blacks, it will be necessary for this area of training to remain very modest”^23^.

The delegate to the General Secretariat feared unrest among the population in his charge. His fears were based on his own interpretation of the “colonial library,” that is the epistemological order that has made it possible to construct Africa as a symbol of otherness and inferiority (Mudimbe, 1988). The mobilization of a racist discourse on the colonized taken in its broadest sense made it possible here to justify a colonial patriarchal view aimed at establishing a hierarchy between movements. However, whether the delegate was convinced of this or whether it was merely a rhetorical justification, this argument was at the very least an effective means of justifying the denial of any symbolic or financial support from the government to European leaders wishing to develop Guiding. While Boy Scouts movements were considered a priority, Girl Guiding was not. And even if the Delegate believed that Guiding would enable the emancipation and protection of young girls, this was not a priority in his view.

Generally speaking, the activities of Girl Guides were largely concerned with hygiene, childcare and “household taste”^24^. In Côte d'Ivoire, young female leaders were encouraged to lead troops of young boys, since “female guardianship (mother, teacher, leader)”^25^ was considered beneficial for small boys. This supervision was viewed as good training for motherhood. Here, the Girl Guides movement was part of the manufacture of a “colonial motherhood” (Hugon, 2004). Guiding was aimed at training girls to become ideal mothers and the pillars of social protection, allowing the colonial authorities to avoid considering the economic or political reasons behind the failures of the few social policies they did have in place. However, training young girls also involved camping and outdoor games, which were considered masculine practices^26^. These activities are indicative of a form of gender blurring. As such, in her work on British Guiding, Mills (2011) has shown that it was such activities, which were traditionally viewed as manly, that legitimized the membership of girls within the Scouting movement, originally intended for boys. However, this blurring was largely reduced and discouraged, particularly through the adaptation of physical practices. Thus, fewer physical tests were required to obtain badges validating progress in colonial Guiding, and outdoor games were often replaced by sewing, singing, or arts and crafts.

The extremely restrictive framework proposed by the French Girl Guides movement in Côte d'Ivoire, aiming at training an ideal docile young African woman, leads us to the hypothesis that, from the point of view of gendered and racialized identities, the colonial Girl Guide movement was even less transgressive than its metropolitan counterpart. Girl Guiding was limited to a secondary space, and primarily valued for its moralizing action. Although its members sometimes carried out military-inspired activities, Guiding focused its educational project on the moral training of young girls, between sexual abstinence, humility, and motherhood, which fitted to the values and activities socially associated with French femininity.

Thus, for two decades, Scouting, supported by the AOF administration, made it possible to train young Ivorian schoolchildren for their future role as auxiliaries. Youngsters voluntarily joined the movement and were gradually trained into an African elite unit, subordinate to the Europeans. Hence, through Scouting, young boys and girls learned norms and values that framed their lives as “ideal colonial subjects” (Lamba, 1985). Girls were to become mothers and educators in charge of their husbands' households, while the latter put themselves at the service of the colonial administration.

## From French to Ivorian Scouting (1945–1969)

From the end of the Second World War, Scouting was challenged in its role of training an elite. While part of the younger Ivorian generation became involved in anti-colonial and independence movements, Scouting was no longer in the vanguard of society. Ivorian Scouting was seeking to redeploy itself to the rhythm of political shifts, while Côte d'Ivoire became autonomous in 1960, under the leadership of President Félix Houphouët-Boigny.

From the 1940s (Cooper, 2002), the Ivorian political landscape was turned upside down. The 1940s and 1950s were marked by the growth of anti-colonial claims. Returning World War II veterans (Mourre, 2017), wealthy African planters—through the *Syndicat agricole africain* (African Agricultural Union) founded in 1944 by Félix Houphouët-Boigny (Cooper, 2014)—and educated young men and women (Barthélémy, 2016) protested against the colonial administration through the newly funded PDCI, which was affiliated to the platform of the *Rassemblement Démocratique Africain* (African Democratic Rally, hereafter RDA) which formulated anti-colonial demands in Côte d'Ivoire. The anti-colonial struggle faced massive repression (Joly, 2015). In this stormy context, Scouting movements reacted in different ways to sedition. Thus, while the Catholic movement remained relatively impervious to sedition, the same cannot be said of the secular movement, marked by the Marxist and anti-imperialist orientations of many Scout leaders, whether they were from metropolitan France or Côte d'Ivoire.

Unlike its religious counterparts, in the 1950s, the secular movement was especially influenced by Marxist readings, whether in metropolitan France or in Côte d'Ivoire. Among these young Frenchmen, we may count the former Resistance fighter and member of the Communist Party René Vautier, who went to Côte d'Ivoire in 1950 (albeit after his Scouting days) to shoot the first French anticolonial film, *Afrique 50* (Nicolas et al., 2015). One may also consider the trajectory of René Dumeste, a young French expatriate for whom Scouting had to allow for an emancipation of young people, just as trade unions allowed for the emancipation of the proletariat. These two trajectories were part of a wider reflection on the colonial future within the secular French Scouting movement (Palluau, 2003).

Several Ivorians leaders also had radical views on colonialism. This was the case of Paul Achy and Jean-Baptiste Pango. The first was a teacher who became a school principal in Port-Bouët (Southern region). He trained in Scouting at William-Ponty school in Dakar^27^. The second was a member of the Youth of the RDA, and attended the World Federation of Democratic Youth Festival (Kotek, 2003) in 1953 in Bucharest^28^. Both were resolutely in favor of the autonomy of Côte d'Ivoire, whether in Scouting or political terms^29^. These two Scout leaders (unlike the Ivorian leaders of the Catholic *Scouts de France*) were not comfortable with the domination of the metropolitan population over their activities. However, beyond these individual lives, as Bancel (2009) has pointed out, this situation should not be viewed as opposing a radically subversive secular Scouting clan to a Catholic Scouting clan allied with the authorities. Most Ivorian leaders occupied more moderate positions between these two extremes.

Beyond these differences, the Catholic Boy Scout movement staked a claim, albeit belatedly, to involvement in African political life. A certain number of publications from the end of the 1950s thus helped to call into question the colonial model. The AOF Catholic Boy Scouts devoted the year 1957 to reflections on the “life of the City”^30^. As in France at the same time, with the emergence of a Catholic Scouting movement close to socialist circles (Prigent, 2011), the idea was to encourage Scouts to discuss citizenship, trade unionism, political ideology (Marxism, capitalism, the social doctrine of the Church), the notion of service, but also the need to practice sports in order to form a healthy mind in a healthy body^31^. Moreover, between 1957 and 1959, columns and editorials aimed at Rovers (teenage boys) questioned the acquisition of citizenship or political autonomy^32^. As for Girl Guides, they were once again placed in the background of these debates (at least in their programmatic form). Their focus was largely once again reduced to and focused on their role as future mothers and wives. Girl Guides had to be “Ready to Serve,” while at the same time fulfilling their “role as mothers and African women”^33^. However, these belated publications from the Catholic Scouting journal were far from subversive. As the process of independence had largely begun, the issue was no longer to take part in subversive activities, but rather to support the process of empowerment within the framework of the Community instituted in 1958 by General De Gaulle (Cooper, 2014). Catholic Scouts were seeking to take over the debate on the future of AOF subjects. From this perspective, they were in tune with the liberal fraction of the colonial administration. Young Africans were in the process of becoming citizens from one year to the next and it was a matter of supporting this movement. This became necessary not only to keep the Scout and Guide movements alive in AOF but also to conform to the primary objective of Scouting, namely the training of an elite.

As the process of independence began, Scouting movements, which were (in theory) supposed to form the vanguard of the people, found themselves left behind, overtaken by anti-colonial and independentist political parties. We rarely find revolutionary ideals either within the secular or Catholic movements (Pango or Vautier are exceptions rather than the rule), but rather the ideal of social progress, of abiding order. Thus, the Ivorian Scouting movements remained pedagogical models of reference for the colonial administration as opposed to the seditious movements of other regions of the Empire, first and foremost Algeria and Indochina (Bancel et al., 2003; Fossard, 2017; Krais, 2018). In Côte d'Ivoire, despite the occasional involvement of some Scouting leaders within Marxist organizations, Boy Scouting and Girl Guiding remained separate from anti-colonial and independence movements.

The empowerment of the AOF Scouting movements in opposition to their supervision by French movements was discussed by AOF Scout leaders from the end of the 1950s until 1960, in debates that echoed those between Léopold Sédar Senghor (the future President of Senegal and supporter of a West-African confederation) and Félix Houphouët-Boigny. However, this autonomy did not mean completely breaking apart from French movements. On the contrary, Ivorian Scouting continued to collaborate with its French counterparts. As such, this tension between autonomy and collaboration was part of the political project of Félix Houphouët-Boigny, who became President of Côte d'Ivoire in 1960. Houphouët-Boigny was a supporter of equal rights between the Ivorians and the French, and, wishing to remain within a French federation, established a system of close cooperation with France (Fauré and Médard, 1982). This resulted in the presence of French *coopérants*^34^ in many areas, particularly in the field of public services dedicated to youth and sports on which Scouting depended (Nicolas, 2019b).

AOF Scouting movements left the French fold between 1957 and 1960. Over the course of 3 years, this independence was debated at length, particularly at a time when Ivorian and Senegalese Scouts opposed autonomist or federalist paths, echoing the positions of Senghor and Houphouët-Boigny. Finally, Scouting organizations officially became autonomous and Ivorian during the summer of 1960 (Nicolas, 2019b, p. 289–290). However, the French continued to be involved in the leadership of the African Scout movements. Thus, in the early 1960s, both the secular Scouts and the Catholic Scouts in Metropolitan France founded institutions that, although they were led by French people, were intended to be “laborator[ies] for inventing a truly African Scouting,”^35^ to use the words of the French secular Scouts. At the same time, development workers employed in the fields of Education or Youth and Sports in Côte d'Ivoire were involved in local Scouting. This was the case of Marcelle Piault, who taught physical education and sports in Ivorian schools. She had been involved in Girl Guiding since the 1950s, and remained so until the 1970s.

As Scouting movements grew, Côte d'Ivoire rapidly became the Mecca of African Scouting. Between the policy of friendship between Côte d'Ivoire and France, and Félix Houphouët-Boigny's desire, as the country's new president, to transform it into a strong regional power (Fauré and Médard, 1982), Côte d'Ivoire was an attractive breeding ground for these continent-wide cooperation movements, which were favorably received by Ivorian political and institutional authorities. Metropolitan secular^36^ Scouts settled in Abidjan in 1968, and Catholic Scouts in 1969. Scouting movements were thus redeployed in the country, adopting positions between nationalist autonomy and the continent-wide metropolitan support that was on offer, all of this against the backdrop of an “Africanization”^37^ of Scouting. In the decades following independence, Scouting movements, supported by the French, grew considerably, with several thousand young Ivorians belonging to the movement by the 1970s. From 1961 onwards, Ivorian Scouting (be it secular, Catholic or Protestant, feminine or masculine) was officially supported by the State. Scouts were heavily subsidized by their parent ministry^38^ and received logistical support through the secondment of popular education and sports officials or through the support of local PDCI members who allowed Scouting troops to use their land or premises. An Ivorian National Scouting College was set up in 1963, bringing together religious and secular Scouts, and unifying the movements in a good relationship with the State^39^. However, tensions soon emerged. The college found it difficult to function due to the historical oppositions between these movements^40^. Moreover, the movements themselves were not free of internal tensions.

For example, between 1960 and 1964, the movement of the *Éclaireurs laïques de Côte d'Ivoire*, led by Simeon Aka N'Wozan and his “glorious team,” undertook to “ivoirise” [to use a much later term (Marshall-Fratani, 2006)] the secular Scouting movement and to exclude the Togolese and Dahomeyans (contemporary Beninese) from it, with the explicit aim of countering the latter's supposed accusations that they viewed Ivorians as “incapable”^41^. This ivoirisation of the secular movement took place against a backdrop of heated debates on the issue of nationality in Côte d'Ivoire from 1963 to 1966, when foreigners from AOF became the scapegoats of the country, and were accused taking jobs from national citizens and monopolizing positions of power (Gary-Tounkara, 2003). Siméon N'Wozan followed this xenophobic line and accused his colleague Paul Achy of favoring “Daho-Togolese” on the one hand and “friends and especially girlfriends who ha[d] no training in Scouting” on the other^42^. N'Wozan assumed that such groups benefited from trips to and training sessions in France, to the detriment of “good” Scouts. By expressing this view, Siméon N'Wozan betrayed a restrictive, nationalistic and gendered definition of what a Scout should be (i.e., male and Ivorian), and suggested in particular that girl Scouts could only be achieving anything by granting sexual favors^43^. After some turbulent years, it was Pelkan Diarra who took over the secular movement between 1969 and 1972—which turned out to be a significant turn of events. Pelkan embodied both proximity to political and bureaucratic power (through his ministerial employment and his status as a member of the PDCI's political bureau), loyalty to the Scouting movement (of which he had been a member for many years) and friendship with French development workers (as evidenced by his close and familiar correspondence with Charles Boganski and René Dumeste).

Thus, just a decade after reaching independence and in spite of a discourse claiming autonomy, the stranglehold of PDCI executives and development workers over the leadership of the Scouting movements was blatant. Moreover, in spite of internal tensions, Ivorian Scouting movements became the preferred space for the implementation of social policies aimed at Ivorian youth. They functioned as a transmission belt between European cooperation projects, youth movements and the social action of the Ivorian State. Ivorian Scouts were to embody Houphouët-Boigny's political projects for the youth of the country.

## Training Ideal Citizens (1960–1979)

In Côte d'Ivoire, Scouting movements appeared to be essential institutions for Houphouët-Boigny's youth policies. Indeed, the President and his comrades at the head of the PDCI delegated a large part of the implementation of popular education policies to Scouting movements^44^. After independence, pedagogical Scouting practices were reformulated in the light of the country's new challenges, particularly the acquisition of a common sense of national pride on the one hand, and participation in building a developed nation on the other. To do so, Scouting and Guiding movements redefined the Scouting syllabus, which now included a patchwork of activities. These were divided into two parts: playful activities (physical activities, discovery of the national territory and supposedly traditional activities) and social and political activities. In this respect, for the PDCI, Scouts at large were a model for young people, just as large sections of Ivorian youth were challenging Houphouët-Boigny's seizure of power.

Scouting aimed to promote the holistic development of the individual—morally, physically, and mentally. In particular, Katrin Bromber and Jakob Krais have pointed out that Scouting's outdoor practices establish a “specific relation of the human to “(national) soil” through self-reliance, physical exposure to hardship, and forming bonds of brother- and sisterhood or solidarity” (Bromber and Krais, 2018, p. 17). Ivorian Boy Scouts took part in physical activities which, following Baden-Powell's model, were inspired by para-military activities: they camped, went on treasure hunts, learned first aid and map reading, made campfires, tied knots, or moorings, trained for self-sufficiency… There was however a degree of novelty involved as more and more sports were included in their range of activities (soccer, volleyball, basketball, athletics), which allowed them to be anchored in a form of modernity that was increasingly defined by urban leisure practices, including sports (Akyeampong and Ambler, 2002). From the 1960s onwards, Girl Guides also became involved in outdoor activities and sports and, as a result, they too incorporated this redefinition of modernity. For example, an Ivorian leader recalls the physical activities practiced in Ivorian Girl Guiding as follows:

“We have track signs, Morse code, how to tie knots. So, we had gatherings like this where you had to show your skill and then we would do it together [girls and boys]. We also made camps. But in the camps, we had sub-camps, with the girls to one side and the boys to the other. But there were certain activities that we did together. [.] At our [girl] level, we had volleyball, basketball. But not soccer. Now the young [girls] are playing soccer. In our time, no one thought a girl could play soccer. No girl of my generation played soccer. But she played volleyball, basketball, running, climbing rope, all that stuff, and we learned how to climb trees and cross rivers”^45^.

The interview with this Girl Guide leader helps to underline once again the symbolic weight of soccer as a male activity. Basketball and volleyball (either through clubs or schools) were female practices that were considered acceptable, and had indeed even been favored by the Abidjan Catholic missions since the 1950s^46^. They were associated with physical training and practices specific to outdoor education (climbing with ropes or trees, river crossings or treasure hunts). Outdoor games, sports and physical training were thus performed by both boys and girls, and, in contrast with the colonial era, while Boy Scouting activities remained more or less alike across time, the landscape of female Scouting underwent important changes. From the 1960s, Girl Guides were widely encouraged to take ownership of outdoor activities and sports. In so doing, girls and boys together appropriated leisure practices that embodied modernity.

In addition to playful physical activities, young people set out to discover their country and visited areas considered representative of the new national territory: remote areas, places of historical significance, nature, historical monuments, and sites of economic development. In this sense, physical activities were also “spatial” activities, as defined by Bromber and Krais (2018, p. 18). Thus, during camps, Scouts and Guides moved around the country and visited new regions, and were encouraged to go beyond their regional or ethnic homeland^47^. Young people also set off to discover places that were significant in terms of their national history. For example, in 1970, a troop of Catholic Scouts went on a treasure hunt in order to locate historic graves from the colonial era^48^. Similarly, in 1971, a troop of Guides investigated the history and social life of a village near their campsite^49^. Young people were also invited to observe nature, inventorying the fauna and flora, creating herbariums or collecting fossils. Finally, Scouts and Guides visited places that symbolized the country's economic development, such as the sites of industrial projects or the buildings of State-owned companies. For example, Scouts and Guides visited the factories of parastatal companies or the offices of *Fraternité-Matin*, the national daily newspaper^50^. These adventures and hikes were not tourism but genuine training, allowing the youths to learn about their national territory. Such learning was meant to highlight modern infrastructure, the natural environment or historical sites. Places of memory, industrial development and natural areas formed a national geography that Scouts and Guides were invited to appropriate, in order to foster a sense of national belonging and patriotism.

At the same time, the range of Scouting activities included cultural activities that were defined as traditional, such as dancing or singing. These practices were part of the enhancement of the country's pre-colonial cultural heritage that was supposed to make it possible to build the post-colonial era. They were at the heart of the Africanization of Scouting. According to their promoters, these activities made it possible to “decolonize individuals and structures”^51^. In concrete terms, this was achieved through role-playing or theatrical performances based on the staging of practices that were presented as local or pre-colonial. These cultural reinventions were freely associated with dances and songs in local or non-local languages. For example, a group from Bingerville (Southern region) could easily sing a Dyula^52^ song without understanding a word of it, as a former Scout reminisced^53^. At the same time, during gatherings, each troop put on dance shows that were specific to their region for each other, in order to showcase local traditions^54^. In so doing, Boy Scouts and Girl Guides were acting in conformity with the objective of intermingling populations that was frequently asserted by Houphouët-Boigny. The mixing of regions and ethnic groups, embodied by national campfire meetings at which local dances and songs were performed, helped to put Scouting right at the heart of the exo-socialization promoted by the Ivorian State.

Playful activities performed by Boy Scouts and Girl Guides, whether they involved discovering the national territory, appropriating a pre-colonial tradition or engaging in sports, were thus aimed at instilling in young Ivorians a sense of national pride, and an attachment to the newly autonomous and independent country and to the achievements of the new president.

In addition, Boy Scouts and Girl Guides were at the forefront of the State's social policies. Indeed, these educated young people, who generally came from urban areas, embodied an ideal form of youth in the new Ivorian nation. At the end of the 1960s, despite the “economic miracle” (Bamba, 2016) that Côte d'Ivoire was experiencing, the country was marked by considerable disparities between the cost of living and wage levels. The slowdown in economic growth led to protest movements, particularly among the youth. These included, between 1967 and 1970, the Bété autonomist movement, led by a student who had returned from France, Kragbé Gnagbé (Gary-Tounkara, 2008, p. 227). At the same time, in 1968, students from the University of Abidjan organized protests (Mbembe, 1985, p. 109–121). Félix Houphouët-Boigny suppressed this student opposition by force (imprisonment, military intervention) and, in 1969, replaced the student union with a newly funded and loyal institution: the *Mouvement des Étudiants et Élèves de Côte d'Ivoire* (Movement of Students and Pupils of Côte d'Ivoire, hereafter MEECI). However, Ivorian students continued to demonstrate against the PDCI, whether in the field of education or employment policies (Gary-Tounkara, 2008, p. 241–242). As Houphouët-Boigny worked actively to subordinate the youth, Scouting movements were closely associated with this process, whether through direct involvement in the ranks of the MEECI and PDCI, or through the implementation of the State's social action policies.

The secular Scouting movement of the *Éclaireurs laïcs de Côte d'Ivoire* was particularly close to the PDCI, as many leaders of the movement joined the ranks of the MEECI during their courses of higher education^55^. In the same way, uniformed Scouting troops provided timely policing services during some official PDCI events^56^. This interference increased during the late 1970s, until some leaders eventually considered officially joining the PDCI, as a youth wing^57^. At the same time, many of the Scout leaders—who were also close to the PDCI and the MEECI—met while conducting fieldwork and became heavily involved in the State's social action missions. These young leaders (who were in their twenties or thirties at the time) and their troops thus directly supported Félix Houphouët-Boigny's desire to bring politicized young people into line.

The Scouts were part of the development programs set up by the Ministry of Youth and Sports. Rather than engaging in political activities, they were encouraged to be of service and, in this context, they strived to set an example for Ivorian youth more widely. They carried out public works: the reconstruction of impassable tracks, building of plant nurseries, providing agricultural aid^58^. They went as far as maintaining public buildings such as town halls or prefectures. These activities were defined by the Ministry as “taking on community responsibilities and the beginnings of training in development practices”^59^. For example, in the 1970s, a troop of secular Scouts from Bondoukou (Northeastern Côte d'Ivoire), led by a teacher, offered literacy programs and did clean-up work in public spaces. They drained the gutters and swept the streets^60^. One could enumerate many examples of such work performed by Boy and Girl Scouts from every movement. The Catholic Scouts thus performed “sweep-up” operations^61^. While Girl Guides did not seem to be involved in explicitly political activities, they were not to be outdone as far as development policies were concerned. For example, in July 1976, the theme of their annual national camps was “community development,” focused on rural Scouting^62^. This emphasis on rural areas is worthy of note, since in the nationalist rhetoric put forward by the Ivorian State, the peasant world became the bearer of progress^63^.

In addition to this subordination to State political movements, Scouting movements were presented as models of probity. Thus, the manufacture of citizenship went beyond the political field *per se* to invest that of morality. The ideal citizen imagined by the PDCI was respectful of moral values. From this point of view, Scouting and Guiding movements thus positioned themselves as exemplary movements, while at the same time providing educational work for the Ivorian population. As such, they took up colonial themes, such as the control of young women's sexuality.

The Girl Guides movement set an example by emphasizing social issues considered to be specifically feminine. A former guide explained that in their pedagogical project, they insisted “on everything that [was] domestic, everything that [concerned] female education, how to prepare the pretty girl, the woman, the home”^64^. Such education was coupled with a “special emphasis on women's empowerment”^65^. While being interviewed in 2016, this retired leader and teacher recalled that during camps, Ivorian Guides learned cooking, childcare, arts-and-crafts and Scouting games. *Le Flamboyant* (a quarterly journal for Girl Guides of all faiths), highlighted this specificity of girls' education. Thus, in 1974, a flower representing the “merry-go-round of activities”^66^ was drawn in the journal in order to showcase activities specific to the social role of the future housewife: childcare, first aid, sewing, cooking, arts-and-crafts (the drawing of the corresponding petal is filled with a bag and a board and not tools). Moreover, the “integral development”^67^ allowed by the Guiding movement presented a corresponding set of skills either deemed specifically feminine (being cheerful, friendly, loving, gracious, and so on) or not (dexterity, physical strength, sports, commitment, responsibility). Thus, if the physical and sporting activities encouraged by the Girl Guides movement had been widened as they appropriated those of the Boy Scout movement, such a moral path particularly echoed the colonial Girl guide movement. The protection of young women in danger stood in opposition to this training. Postcolonial Girl Guiding—like its colonial ancestor—was conceived for the moral protection of young girls in urban areas. Henceforth, the main partners of the Girl Guides movements in the Ivorian State were women's shelters^68^. Mainly located in urban areas from the Central and Southern regions, they welcomed young women in urban areas in order to protect them. The migration of “little nieces and little maids” (Jacquemin, 2012) from rural outskirts to urban areas was a constant concern for educators and leaders, who viewed cities as spaces of perversion that endangered the moral fiber of Ivorian girls, where they would escape from the control and protection of their families.

Boy Scouts also took part in this moral training, in particular as both Boy Scouts and Girl Guides set up moralization campaigns stigmatizing undesirable behaviors. This included the organization of theatrical performances. The sketches dealt with hygiene, morality, respect for elders, the importance of schooling and help for the elderly. The bad behavior of young people was targeted as “defects of society”^69^. For example, a now retired teacher from the North-Eastern region, who led a troop in the outskirts of Bouna (North-Eastern Côte d'Ivoire) in the 1970s reminisced on the educational and moralizing virtues of Scouting theater as follows:

The Scouts “acted out the household scenes, the maids who go to Abidjan, in the big cities, the consequences of this, unwanted pregnancies, et cetera. People perform this in the villages. And when they perform it, the whole village insists on coming to see it, eh! The defects of society, for example: it's all bad. It has often helped a lot in some villages. Some chiefs even encourage it!” ^70^

The performances given by the Scouts promoted an image of young Scouts concerned with integrity and work to be set against a deviant, disobedient and lazy youth, prone to rural exodus and to an overly-precocious sexuality (here targeting young girls in particular). At the same time, during shows attended not only by other Scouts or Guides, but above all by the surrounding population (parents, neighbors, young people), the sketches made it possible to “show the population [that the Scouts were] not bandits, that they [were] people who can serve and can be trusted,” as another retired teacher, a former leader of a group of secular Scouts in Bondoukou, pointed out^71^.

Indeed, while the Scouting movements were institutions that attempted to guarantee the moral probity and subordination of Ivorian youth, the behavior of their members did not always reflect these aims. Contrary to the aforementioned desires of their leaders, troops could at times engage in begging or petty crime^72^. It thus appears that, while Scouting movements generally seemed to align themselves with hierarchical demands for obedience, young Scouts did not always comply with these demands. For example, in 1968, a group of secular Scouts invited to a camp that had been organized with pomp and circumstance by French and Ivorian Scouting authorities, under the good auspices of the PDCI, stood accused of being “selfish, willingly cranky and pretentious” in one case, “filled with schoolboy derision” in another or even prone to exerting “a detestable influence on those around him, impulsive and vulgar”^73^ in the last. From this point onwards, these groups of young high school students, teachers, clerks and craftsmen (all aged between 17 and 27 years old), instead of showcasing the good morals of Ivorian Scouting, embodied the cracks in the moralization program that was being implemented by Scouting authorities in cooperation with the Ivorian government. Furthermore, European leaders put great stock in the impact of Scouting as a source of salvation, particularly in terms of sexual abstinence as far as Girl Guiding was concerned. However, their position did not necessarily extend its influence to all girls. In this regard, the letters sent by young women from the Swiss Guiding movement (based in Côte d'Ivoire between 1968 and 1975) to their parent federation in Switzerland are particularly illuminating. The Swiss leaders appeared convinced of the moral and religious^74^ significance of Christian Scouting. However (echoing their French predecessors from the 1940s), they deplored the harmful atmosphere of the metropolis, using the Christian rhetoric of sin and the Fall:

“In Abidjan, [a girl club house] would be particularly appropriate given the number of girls who arrive there – all naïve – from their village. They are disoriented, lost in the big city and poorly supported by distant parents or a guardian. They do stupid things and get lost”^75^.

Here, they particularly target young leaders of both the Protestant and Catholic girl movements who got pregnant out of wedlock. Beyond purely religious considerations, these pregnancies had major social consequences for the young women. For example, in 1973, a leader who was supposed to take over the running of the infirmary of the Unionist Girl Scouts Association became pregnant. According to the Swiss leader, the young woman had to leave the hospital where she worked and was not allowed to return to her family, nor was she allowed to take on the responsibilities that the leaders in the Girl Guiding Movement had planned for her. On this occasion, the Swiss leaders placed particular emphasis on “the difficulty of making girls aware of their responsibilities”^76^. However, these pregnancies may be questioned in light of the gendered social relations that were upheld within the Ivorian Scouting Movements themselves. In this respect, one letter from a Swiss leader allows us to shed new light on the sweep-up operations during which loyalty to the Independent State and Scouting's civic training were performed. The Swiss leader recounts that when girls and boys carried out a cleaning operation in the Treichville and Adjamé neighborhoods with the collaboration of the Abidjan garbage service in 1975, “Our 200 girls were a bit shaken up by the 1,000 guys happy to have the chance to command them!”^77^ We do not have any more details about the exact manner in which the girls were “shaken up.” However, this opens up avenues for inquiry. During this event, young Boy Scouts once again deviated from the virtuous path imagined for them and behaved aggressively toward the girls. We thus need to pay particular attention to the redeeming agenda of youth movements more generally, in particular in terms of the way they emphasized the individual responsibility of girls on the one hand, and religious morality on the other. Such themes—which were dear to the Scouting movement—need to be cross-read in light of the history of discursive, physical and sexual violence faced by girls.

Thus, in all Scouting movements, despite a marked political loyalty, there were gaps emerging between the project of training a social elite, as put forward by the leadership of the movement, and the implementation of this project, particularly from the point of view of the moral training of young people. This tension was reflected in the programs organized by Scouting leaders, from sweep-up operations to theater plays. The Scouts implemented the Ivorian youth moralization program, but they were also supposed to promote morals and discipline through example, performing the role of a bulwark against immorality—despite their own behavior often being at odds with these aims. Nevertheless, by asserting the position of a minority fraction of the youth within Ivorian society, Scouting movements supported the elite position of an idealized, educated and uniformed younger generation that supposedly had little interest in opposing the president.

## Conclusion

Somewhat provocatively, John Lonsdale wrote in 1986 that “much was excitedly expected of the successor African countries. Their civilizing mission did not seem so very different from what had gone” (Lonsdale, 1986, p. 154). Indeed, in many respects, many independent governments perpetuated the civilizing mission of their former colonizers. As far as youth education was concerned in Côte d'Ivoire, strong lines of continuity may be drawn with colonial themes. On the one hand, attention to the sexual behavior of young women and to moral salvation in general became an important theme of colonial governance from the 1940s onwards and was perpetuated in the following decades, as the State implemented a form of coercive welfarism (Fourchard, 2018). On the other hand, the pedagogical principles of Scouting—which made it possible to establish the political subordination of an elite fraction of youth—were encouraged both by the political administration of the AOF and then by Félix Houphouët-Boigny.

Ivorian Scouting movements thus became places where a model citizenship was being manufactured: a model citizenship steeped in patriotism and morality, respectful of hierarchies and order, and to which undesirable behaviors, ranging from immorality to political sedition, were to be opposed. Such opposition and instrumentalization was not the sole privilege of Côte d'Ivoire, as Bromber and Krais (2018) have recently pointed out. However, the case of Côte d'Ivoire allows us to further highlight the cross-cutting nature of this process of training an elite fraction of youth, particularly in a non-Socialist, non-revolutionary State. Moreover, it makes it possible to underline the extent to which these New Men may have been politically subordinated, particularly when their training was based on a youth movement such as Scouting.

In line with the “parental reading of political subordination”^78^ offered by Mbembe (1985, 14–16) and more recently taken up by Havard (2009), we may indeed understand the Scouts as the model of a filial citizenship, subject to the Father of the Nation. However, the desire of the independent Ivorian government to incorporate youngsters under a single, unambiguous, and subordinate collective identity came up against the dissent displayed by these same youngsters. Whole sections of the youth—such as students—took upon themselves to protest the political stranglehold of the PDCI and failed to submit to the codes of morality promoted by the State and the Scouting movements. And even members of the Scouting movements eventually took part in such dissent. One way to see this tension emerge is through the gender studies approach, as set forward in this article. It allows us to highlight the focus of Scouting movements on the moral salvation of young girls. In this perspective, “girling the subject” (George, 2014, p. 1) of Scouting and Sports proves helpful when it comes to analyzing the State's will and (in)ability to control the morals of young citizens. Finally, this case study, at the cross-roads of Gender Studies, Sport History and African History, further calls for expanding such perspective beyond Sub-Saharan Africa, in order to understand the ambiguous roles played by sporting youngsters within the political manufacture of the subordination of young people.

## Data Availability Statement

The raw data supporting the conclusions of this article will be made available by the authors, without undue reservation.

## Author Contributions

The author confirms being the sole contributor of this work and has approved it for publication.

## Conflict of Interest

The author declares that the research was conducted in the absence of any commercial or financial relationships that could be construed as a potential conflict of interest.

## Footnotes

^1^“Dans mon milieu, les scouts, la grande majorité c'est les enfants des fonctionnaires. Parce que d'abord ils sont bien habillés. Ils marchent au pas. Ça donne l'impression de l'ordre, la discipline. Donc chacun était fier de voir son enfant dans ces rangs-là !” Anonymous interview with a former Boy Scout (*Éclaireurs laïques de Côte d'Ivoire*), 4 April 2016, Bouaké.

^2^I wish to thank Jakob Krais, my colleagues from the Université de Lausanne and *Frontiers in Sports and Active Living*'s anonymous reviewers for generously commenting on the present paper. I also thank Kate McNaughton for thoroughly proofreading the text.

^3^The French colonies of West Africa were gathered together into a single administrative entity, the Afrique Occidentale Française. These colonies were Côte d'Ivoire, Dahomey (now Benin), Guinea, Mauritania, Niger, Senegal, French Sudan (now Mali), Upper Volta (now Burkina Faso), and Togo.

^4^This elimination follows the “false conspiracy” affair, during which he also dismissed early independence fighters threatening his authority (Diarra, 1997).

^5^This protest was part of a general protest movement among African students during the 1960s (Blum et al., 2016).

^6^“Socialization dans une assignation politique subordonnée”.

^7^Based on cross-referencing archives and interviews with former Scouts and Guides.

^8^“Scoutisme français. Origines du scoutisme en Côte d'Ivoire,” *Bulletin de la Côte d'Ivoire*, 1st January 1948. Copies of the *Bulletin* were browsed at the *Archives nationales d'Outre-mer de France* in Aix-en-Provence.

^9^“Scoutisme français. Origines du scoutisme en Côte d'Ivoire,” *Bulletin de la Côte d'Ivoire*, 8 January 1948.

^10^Letter from Ousmane Thiané Sar (secular Scout leader) to Obed Yansunni, 15 March 1953. Val-de-Marne Archives (hereafter VDM), 542J 420.3.

^11^“Scoutisme français,” *Bulletin de la Côte d'Ivoire*, 8 January 1948.

^12^Fédération éclaireuse suisse, *Amitié internationale Côte d'Ivoire*, 1968, p. 17. Swiss Federal Archives (hereafter SFA), J2.334-01 2008/3 (242).

^13^Less than 10,000 young Ivoirians (among whom less than 2,000 girls) were sent to school in the late 1930s (Désalmand, 1983, p. 267–268).

^14^“Donne ainsi pratiquement un magnifique exemple de l'unité française, [mais] il reste cependant un mouvement d'élite réservé à la formation des chefs.” In *Service de la Jeunesse de l'Afrique noire, Instructions générales aux chefs locaux*, n°1,1943, p. 9. SNA, O 516 (31).

^15^“Une véritable fraternité, où nous jouons le rôle de frères ainés, naît entre scouts blancs et noirs servant ensemble un même idéal. C'est une fraternité hiérarchisée au sommet de laquelle rayonne notre patrie, la France.” In Letter from F. Berthet (Director General of Political, Administrative and Social Affairs) to the Director General of Public Education and Sports), 3 October 1942. Senegal National Archives (hereafter SNA), O 2 (31).

^16^“Instincts ataviques de leur race.” In “Scoutisme français. Pour les jeunes et leurs parents,” *Bulletin de la Côte d'Ivoire*, 18 December 1947.

^17^“De ceux qui savent promettre et se montrer capables de tenir leurs engagements.”

^18^“Multiples services.” In Dorothé Akpovo, “Côte d'Ivoire,” *Lettre de la province. Bulletin de l'association des scouts de France, province d'AOF—Togo*, March 1956.

^19^Brother Jean-Pierre Lintanf, “Pour une vraie,” *Scout AOF*, n°1, 1957.

^20^Christian Kuawu, “Le jeune homme et l'amour”, *Lettre de la Province. Bulletin de l'association des Scouts de France, province d'AOF—Togo*, April 1956.

^21^“l'atmosphère fiévreuse et peu morale des grandes villes” and “restaurants féminins et une surveillance des rues en même temps qu'une propagande de moralité.” In Mrs D'Autheville, Claudet, Bourgoin, Ledran, Matte and Mallet, “Sous-commission féminine,” *Rapport général sur la Conférence africaine de la jeunesse*, 3–5 February 1943. SNA, O 87 (31).

^22^Anonymous interview with a former Boy Scout (*Éclaireurs laïques de Côte d'Ivoire*), 4 April 2016, Bouaké.

^23^“d'émanciper que très lentement [les femmes] et il ne serait pas question, à part les exceptions qui confirmeront la règle, de formation parallèle à celle des garçons, ainsi que nous la concevons. La femme étant aux yeux des Noirs le moyen de travail et l'instrument qui perpétue la vie, il sera nécessaire de rester très humble dans ce domaine de formation” In *Programme d'action du délégué du secrétariat général de la Jeunesse en Afrique occidentale française, circa* 1944. SNA, O 515 (31).

^24^“le goût ménager” In Service de la Jeunesse de l'Afrique noire, *Instructions générales aux chefs locaux*, n°1, 1943, p. 26. SNA, O 516 (31).

^25^“la tutelle féminine” In “Le mouvement scout de France,” *Lettre de la province. Bulletin de l'association des scouts de France, province d'AOF—Togo*, October–November 1955.

^26^Guides de France, “La vie en Outre-Mer en 1958–59,” *Journées nationales de commissaires de district et aumôniers diocésains*, Paris, 1959, p. 6. Youth and Sports National Institute of Côte d'Ivoire Archives (hereafter INJS), “Marcelle Piault” collection, file “mouvements de jeunesse.”

^27^Letter from René Dumeste to the French Scouting Headquarter, 24 July 1954. VDM, 542J 420.3.

^28^Letter from Paul Achy to René Dumeste, 10 December 1954. VDM, 542J 420.3.

^29^Ibid.

^30^“La vie de la cité” In “Plan d'année,” *Scout AOF*, n°1, 1957.

^31^Ibid.

^32^“Faire des hommes libres,” *Scout AOF*, n°2, 1957; Jacques Bonnamour, “Former des hommes au service de l'Afrique,” *Scout AOF*, n° 6, 1959.

^33^“Rôle de mères et de femmes africaines” In “Ta cité,” *Scout AOF*, n°1, 1957; B. Enreille, “Après Bamako,” *Lettre de la province. Bulletin de l'association des scouts de France, province d'AOF—Togo*, May 1957.

^34^“*La Coopération”* was a system of partnerships ostensibly aimed at redefining Franco-African relations after decolonization, notably through “technical assistance” programs.

^35^“Laboratoire[s] pour inventer un scoutisme proprement africain” In Collège africain et malgache des Éclaireurs laïques, “Organization,” 1965. VDM, 542J 495.

^36^“Secular” is used here as a translation of “*laïque*,” which in the context of Scouting referred to movements that were non-denominational but not necessarily non-religious, and certainly not atheist; on the contrary, in the period following independence, *laïque* Scouting groups tended more to be ecumenical, and in particular were often the only groups that would accept Muslim members.

^37^It was no longer just a question of replacing metropolitan executives with African executives, but also of promoting a discourse on the adaptation of Scouting to the African context through the demand for practices that were defined as traditional.

^38^At this date, the only organizations that were approved and financially supported by the State were either close to the PDCI or of Western heritage (Scouting, Catholic or Protestant associations, sports associations, reading groups). Regional cultural associations were almost completely deprived of State subsidies, as were Muslim groups or groups of divergent political affiliation. Scouting movements received relatively large subsidies, compared to sports federations, which had a much larger membership. For example, in 1965, Scouting movements received 2,300,000 CFA for the year, including 300,000 CFA for each men's movement. Meanwhile, sports federations received between 600,000 and 800,000 CFA per minor sports federation (volleyball, judo, basketball, handball, or cycling). In view of the membership numbers of the respective groups (much higher for the sports concerned), the political support shown by the government for Scouting movements is remarkable. *Official Gazette of Côte d'Ivoire*, 1961–1979. The country's official journals are kept in the National Archives of Côte d'Ivoire.

^39^Survey filled out by the secular Scouting movement of Côte d'Ivoire and sent to the French Secular Scouting Overseas department, 1963. VDM, 542J 1202.

^40^Letter from Charles Boganski to Pascal Assoko, 3 December 1969. VDM, 542J 1202.

^41^“équipe glorieuse” et “incapables.” In Minutes of the funding Congress the *Éclaireurs laïques de Côte d'Ivoire*, 2 July 1960. VDM, 542J 1202.

^42^“Daho-Togolais” and “copains et surtout des copines n'ayant aucune base de formation scoute.” In Letter from Siméon N'Wozan to Charles Boganski, 29 April 1964. VDM, 542J 1202.

^43^This may be analyzed in line with Broqua et al. (2014)'s concept of sexual-economic exchange, borrowed from the Italian anthropologist Paola (Tabet, 2004). Their analysis of “transactional sexuality” highlighted how sexuality, gender and economic status were intertwined in twentieth Century Africa. In this perspective, Vidal's paper (1977) on the “Sex war” held in Abidjan in the 1970s is enlightening. She showed to which extent marriage and relationships were framed by monetary issues, in the context of the emergence of a “small urban bourgeoisie.”

^44^Similarly, the parallel attempt of the Ivorian civic service, founded in the early 1960s, was considered a failure (Guez, 1981; Mbembe, 1985, p. 95; Nicolas, 2019b, p. 282–284). In this regard, I wish to thank Daniel Heller for sharing his knowledge of the history of the Ivorian civic service.

^45^“On a les signes de piste, le morse, comment faire les nœuds… Donc, on a des rassemblements comme ça où il fallait montrer son adresse et là, on faisait ensemble [filles et garçons]. On faisait aussi les camps. Mais dans les camps, on avait des sous-camps, avec les filles à part et les garçons à part. Mais il y avait certaines activités qu'on faisait ensemble. […] À notre niveau [féminin], on avait le volleyball, le basket. Mais pas le foot. C'est maintenant que les jeunes [filles] pratiquent le foot. À notre époque, personne ne pensait qu'une fille pouvait faire du foot. Aucune fille de ma génération ne faisait de foot. Mais elle faisait le volley, le basket, la course, grimper à la corde, tout ça et cætera. on apprenait à grimper aux arbres à traverser les rivières”. In Anonymous interview with a former Girl Guide (*Guides de Côte d'Ivoire*), 3 March 2016, Abidjan.

^46^The lack of physical contact and the fact that these sports have historically been played indoors make them sports historically associated with women (Nicolas, 2019b, p. 111).

^47^Anonymous interview with a former Boy Scout (*Scouts catholiques de Côte d'Ivoire*), 20 February 2016, Abidjan.

^48^Joseph Bilé Bilé, “rapport sur Assikoi,” 16 March 1970. VDM, 542J 1202.

^49^Isabelle, “Oumé,” *Le Flamboyant*, n°1, 1971, p. 12. Copies of *Le Flamboyant*—Ivorian Girl Guides' journal in the 1970s—are preserved at the INJS: “Marcelle Piault” collection, file “mouvements de jeunesse.”

^50^Véronique Agbaya and Joséphine Djoe, “Rapport du camp de formation des cheftaines éclaireuses unionistes de Côte d'Ivoire,” *Le Flamboyant*, n° 6, 1973–1974.

^51^“décolonizer les individus et les structures” In Collège africain et malgache des Éclaireurs laïques, “Organization,” 1965. VDM, 542J 495.

^52^Generally speaking, Dyula is mainly spoken by Northerners, though this is something of a simplification (Bouquet, 2003).

^53^Anonymous interview with a former Boy Scout (*Éclaireurs laïcs de Côte d'Ivoire*), 18 February 2016, Abidjan.

^54^Anonymous interview with a former Boy Scout (*Éclaireurs laïcs de Côte d'Ivoire*), 2 April 2016, Bondoukou.

^55^Anonymous interview with a former Boy Scout (*Éclaireurs laïcs de Côte d'Ivoire*), 18 February 2016, Abidjan.

^56^Anonymous interview with a former Boy Scout (*Éclaireurs laïcs de Côte d'Ivoire*), 4 April 2016, Abidjan.

^57^*Ibid*.

^58^Such focus on youth work may also be found in Senegal (Tiquet, 2016) or Ghana (Nicolas, 2017).

^59^“Prise de responsabilités communautaires et des amorces de formation aux pratiques du développement” In Lamine Ba, “Note de synthèse des questions étudiées à Abidjan,” In *Premier séminaire sur le sport*, 1974. Ministry of Youth and Sports archives, “Miscellaneous” collection.

^60^Anonymous interview with a former Boy Scout (*Éclaireurs laïcs de Côte d'Ivoire*), 31 March 2016, Bondoukou.

^61^“Coup de balai” In Anonymous interview with a former Boy Scout (*Scouts catholiques de Côte d'Ivoire*), 1 March 2016, Abidjan.

^62^“Développement communautaire” In Madeleine Boti, “Rapport d'activité 76–77,” 1 November 1977. INJS: “Marcelle Piault” collection, file “mouvements de jeunesse.”

^63^Félix Houphouët-Boigny, himself a former planter, places particular emphasis on the advent of a “*bourgeoisie de planteurs*” who would spearhead the nation (Gastellu and Affou Yapi, 1982).

^64^“Sur tout ce qui est ménager, tout ce qui est éducation féminine, comment préparer la jolie fille, la femme, le foyer” In Anonymous interview with a former Girl Guide (*Guides de Côte d'Ivoire*), 3 March 2016, Abidjan.

^65^“Accent particulier sur la prise de responsabilité des femmes” In *Ibid*.

^66^“Ronde des activités” In Le scoutisme féminin assure le développement intégral de la jeune fille,” *Le Flamboyant*, 1974, pp. 10, 14.

^67^“Développement intégral” In *Ibid*.

^68^These are mainly located in the South and Center of the country, the most urbanized areas: 24 homes in the southern region and 20 in the central region, compared with 5 in the eastern region, 5 in the central-western region, 9 in the West and 5 in the North (Kouassi-Kouadio, 1979, p. 124–126).

^69^Anonymous interview with a former Boy Scout (*Éclaireurs laïcs de Côte d'Ivoire*), 2 April 2016, Bondoukou.

^70^“Jouaient les scènes de ménage, les bonnes qui vont à Abidjan dans les grandes villes, les conséquences que ça engendre, les grossesses indésirées, et cætera. Les gens jouent ça dans les villages. Et quand ils jouent tout le village insiste pour venir assister hein ! Les tares de la société par exemple: tout ça là, c'est pas bon, là. Ça a beaucoup aidé souvent certains villages. Et il y a même des chefs qui encouragent !” In *Ibid*.

^71^“Montrer à la population [que les scouts ne sont] pas des bandits, que [ce sont] des gens qui [peuvent] servir et que on [peut] avoir confiance.” In Anonymous interview with a former Boy Scout (*Éclaireurs laïcs de Côte d'Ivoire*), 31 March 2016, Bondoukou.

^72^Letter from Pelkan Diarra to Charles Boganski, 12 January 1970. VDM, 542J 1202.

^73^Assessment of the behavior of members of the *Éclaireurs laïques de Côte d'Ivoire* during the 6th session of the annual African Training Camp organized in Abidjan, 1 February 1968. VDM, 542J 1202.

^74^One of the first leaders who left for Côte d'Ivoire even decided to take holy orders. Letter from Wilhelmine Burgat to Maïse Montandon, 19 April 1969 [AFS: J2 334.01].

^75^“À Abidjan, ce serait particulièrement indiqué vu le nombre de filles qui y arrivent toutes naïves de leur village. Elles sont désorientées, perdues dans la grande ville et mal soutenues par de lointains parents ou un tuteur. Elles font des bêtises et se perdent.” Letter from Wilhelmine Burgat to Maïse Montandon, January 1969 [AFS: J2 334.01].

^76^“La difficulté à rendre les filles conscientes de leurs responsabilités.” Report, May 1973 [AFS: J2 334.01].

^77^“Nos 200 filles ont été un peu bousculées par les 1,000 gars heureux de pouvoir les commander !” Letter from Corinne Vuilleumier to the Fédération éclaireuse suisse, 28 February 1975 [AFS: J2 334.01].

^78^“Lecture parentale de la subordination politique.”
